# Stabilizing Bicontinuous Emulsions with Sub‐Micrometer Domains Solely by Nanoparticles

**DOI:** 10.1002/advs.202406223

**Published:** 2024-08-20

**Authors:** Alessio J. Sprockel, Tessa N. Vrijhoeven, Henrik Siegel, Ffion E. Steenvoorden, Martin F. Haase

**Affiliations:** ^1^ Van't Hoff Laboratory for Physical and Colloid Chemistry Department of Chemistry Debye Institute for Nanomaterials Science Utrecht University Utrecht The Netherlands

**Keywords:** bicontinuous emulsions, bijels, colloidal particles at interfaces, nanomaterials, phase separation

## Abstract

Nanoparticle‐stabilized, bicontinuous interfacially jammed emulsion gels (bijels) find potential applications as battery, separation membrane, and chemical reactor materials. Decreasing the liquid domain sizes of bijels to sub‐micrometer dimensions requires surfactants, complicating bijel synthesis and postprocessing into functional nanomaterials. This work introduces surfactant‐free bijels with sub‐micrometer domains, solely stabilized by nanoparticles. To this end, the covalent surface functionalization of silica nanoparticles is characterized by thermogravimetric analysis, mass spectrometry, Fourier‐transform infrared spectroscopy, and contact angle measurements. Bijels are generated with the functionalized nanoparticles via solvent transfer induced phase separation (STrIPS), enabling the optimization of nanoparticle functionalization and surface ionization. Nanoparticles of intermediate functionalization and controlled negative surface charge stabilize bijels with sub‐micrometer liquid domains. This remarkable control over bijel synthesis provides urgently needed progress to facilitate the widespread implementation of bijels as nanomaterials in research and applications.

## Graphical abstract

Bicontinuous interfacially jammed emulsion gels (bijels) are versatile nanomaterials with tunable properties, ideal for applications in catalysis, energy storage, and membrane separations. This study presents surfactant‐free bijels with sub‐micrometer domains, stabilized exclusively by nanoparticles. The resulting bijel structures can be precisely adjusted through covalent functionalization and ionization of the nanoparticles.

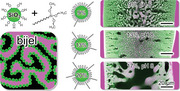

## 1 Introduction

Particle‐stabilized emulsions (PSEs) are important constituents in foods,^[^ ^1^ ^]^ cosmetics,^[^ ^2^ ^]^ pharmaceuticals,^[^ ^3^ ^]^ paints and coatings.^[^ ^4^ ^]^ In comparison to surfactant stabilized emulsions, PSEs have exceptionally high stability against coalescence,^[^ ^5^ ^]^ and they can consist of environmentally friendly components.^[^ ^6^ ^]^ However, unlike surfactants, most untreated particles are unable to stabilize emulsions because they strongly prefer the water or the oil phase over the interface. For example, ceramic particles made of silica, alumina, or titania have oxidized surfaces, rendering them hydrophilic without significant interfacial activity. To obtain interfacially active and emulsifying particles, surface functionalizations with organic molecules are often required.

The functionalization of particles can be realized by covalently attaching hydrophobic groups on their surfaces.^[^ ^7^ ^]^ Alternatively, the physisorption of ionic amphiphiles,^[^ ^8^, ^9^ ^]^ multivalent ions,^[^ ^10^ ^]^ or polyelectrolytes^[^ ^11^ ^]^ can modulate the interfacial activity of the particles. Moreover, the degree of ionization of surface groups influences the particle's interfacial behavior.^[^ ^12^ ^]^ Combining these knowledge gains enables control over emulsion type, facilitating catastrophic phase inversions from oil‐in‐water to water‐in‐oil emulsions.^[^ ^13^ ^]^ Intriguingly, at the transition of this inversion, the particles are able to stabilize oil/water interfaces with both positive and negative Gaussian curvature.^[^ ^14^, ^15^ ^]^ This important insight has contributed to the discovery of bicontinuous interfacially jammed emulsion gels (bijels).^[^ ^16^, ^17^ ^]^

Bijels are uninterrupted networks of two interwoven liquids that are separated by a semipermeable layer of particles.^[^ ^18^ ^]^ Each of these three continuous components has distinct properties, enabling the combination of three materials with tunable attributes into a single volume. Due to their tri‐continuous architecture, bijels are useful for the simultaneous transport of electricity, chemicals, and heat in batteries,^[^ ^19^ ^]^ filtration membranes,^[^ ^20^, ^21^ ^]^ and chemical reactors.^[^ ^22^ ^]^ Bijels are stabilized via interfacial jamming of surface‐functionalized particles. However, stabilizing bijels requires precise control over surface functionalization, a challenge that still impedes widespread implementation of bijels in research and applications.

Successful bijel stabilization has been achieved by surface functionalizing particles in situ with ionic surfactants.^[^ ^23^, ^24^, ^25^, ^26^ ^]^ But, when surfactants are used to functionalize the particles, bijel synthesis becomes a simultaneous interplay of multiple chemicals, restricting control and analytical understanding of the associated non‐equilibrium self‐assembly process. Covalently functionalized particles can enhance the reproducibility of bijel synthesis, and simplify the chemistry needed to postprocess bijels into functional nanomaterials. Such covalent surface functionalizations of particles have been introduced, for example by employing 3‐(aminopropyl) triethoxysilane (APTES),^[^ ^17^, ^27^ ^]^ hexamethyldisilazane (HDMS),^[^ ^28^, ^29^ ^]^ and 3‐(Trimethoxysilyl) propyl acrylate (TPA).^[^ ^30^, ^31^ ^]^ Nevertheless, covalently functionalized particles have not yet been shown to enable the stabilization of bijels with sub‐micrometer domains, an attribute important for the applications of bijels as nanomaterials.

Here, we introduce the synthesis of bijels with sub‐micrometer domains with nanoparticles of well‐defined covalent functionalizations and surface ionizations. To this end, we functionalize silica nanoparticles (SNPs) with alkyltriethoxysilanes (ATEOS) to obtain ATEOS‐SNPs. The quantitative ATEOS functionalization is proportional to the reactant amount during synthesis, as quantified by Fourier‐transform infrared spectroscopy (FTIR), thermogravimetric analysis (TGA), and contact angle measurements. We employ these well‐characterized particles to synthesize ATEOS‐SNP stabilized bijels via solvent transfer‐induced phase separation (STrIPS).^[^ ^26^, ^32^ ^]^ Confocal microscopy analysis demonstrates control over bijel structure by gradually varying the extent of ATEOS‐SNP functionalization and surface ionization. While prior reports have described bijels with sub‐micrometer domains by combining nanoparticles with surfactants in situ,^[^ ^24^, ^26^, ^33^ ^]^ the control over bijel synthesis attained here demonstrates for the first time sub‐micron domain bijels with covalently functionalized particles, introducing nanomaterials with tunable and functional attributes.

## 2 Results and Discussion

**Figure 1:**
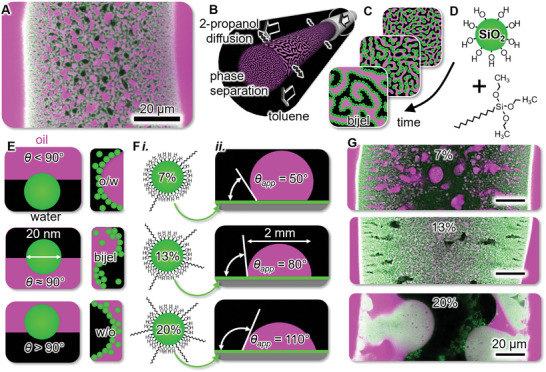
Bijel synthesis and silica nanoparticle (SNPs) functionalization. A) Confocal laser scanning microscopy (CLSM) image of a bijel fiber segment with oil (magenta), water (black), and SNPs (green). B) Schematic of bijel fiber fabrication via solvent transfer induced phase separation (STrIPS). C) Illustration of interfacial particle self‐assembly. D) Illustration of SNPs and dodecyltriethoxysilane (DoTEOS). E) Schematic of the effect of the three‐phase contact angle *θ* on interfacial curvature and the emulsion type (oil‐in‐water: o/w, bijel, water‐in‐oil: w/o). F*i*.) Depiction of increasing %‐DoTEOS functionalization (7%, 13%, 20%) of SNPs and F*ii*.) Measurements of the apparent contact angle *θ*_app_ of sessile diethylphthalate (DEP) droplets on DoTEOS‐SNP films in water. G) CLSM images of bijel fiber sections fabricated with SNPs of increasing %‐DoTEOS functionalization at pH 8, see also Figure 5C*i*).

**Figure** **1**A shows a confocal laser scanning microscopy (CLSM) image of a short section of a much longer bijel fiber. The bijel fiber is surrounded by fluorescent oil, displayed in magenta color. Within the fiber, the oil is interwoven with black‐colored water. On the interface between water and oil is a rigid film of green‐colored silica nanoparticles. Figure 1B depicts the synthesis of the bijel fiber via solvent transfer‐induced phase separation (STrIPS). During STrIPS, a mixture of oil, water, and 2‐propanol is flown into toluene. 2‐propanol diffuses into toluene, triggering phase separation and self‐assembly of the nanoparticles onto the oil/water interface, arresting the bicontinuous fluid arrangement as schematically depicted in Figure 1C. In this work, silica nanoparticles self‐assemble on the oil/water interface due to their surface functionalization with alkyltriethoxysilanes (ATEOS), as depicted in Figure 1D.

Bijel stabilization requires control over the three‐phase contact angle (*θ*) of colloidal particles. By changing from *θ* < 90° to *θ* > 90°, particle‐stabilized emulsions undergo phase inversion from oil‐in‐water (o/w) to water‐in‐oil (w/o) droplets.^[^ ^34^ ^]^ Only particles with *θ* close to 90° can simultaneously stabilize both the positive and the negative Gaussian curvatures of the oil/water interface in a bijel, as schematically depicted in Figure 1E.^[^ ^35^ ^]^

In this work, we test the hypothesis that for ATEOS functionalized silica nanoparticles (ATEOS‐SNPs), *θ* can be controlled by the number and chain length of covalently attached ATEOS molecules. Figure 1F *i* illustrates ATEOS‐SNPs with an increasing number of covalently attached dodecyltriethoxysilane (DoTEOS) moieties (DoTEOS‐SNPs). As the basis for normalizing the %‐DoTEOS functionalization, the calculated number of silanol groups on the particles is used. Figure 1F *ii* illustrates the dependency of the apparent contact angle *θ*_app_ on the %‐DoTEOS functionalization. We determine *θ*_app_ for sessile droplets of diethylphthalate (DEP) on thin films of DoTEOS‐SNPs in water. As the %‐DoTEOS functionalization is increased from 7% to 20%, *θ*_app_ increases from 50° to 110°.

Figure 1G shows CLSM images of bijel fiber sections synthesized via STrIPS with DoTEOS‐SNPs of 7% to 20%‐functionalization. At 7%‐functionalization, magenta oil‐in‐water droplets are observed, with green particles primarily located in the black water regions of the fiber. For 20%‐functionalization, bottom image), the structure is strongly coarsened with green DoTEOS‐SNPs primarily overlapping with the magenta oil regions in the fiber. Remarkably, at 13%‐DoTEOS functionalization (middle image), we observe a stable bijel composed of sub‐micrometer oil and water networks. These initial results qualitatively agree with the schematic picture in Figure 1E. Moreover, the measurements of *θ_app_* in Figure 1F *ii* indeed suggest a transition from hydrophilic to hydrophobic DoTEOS‐SNPs as %‐functionalization is increased. In the following manuscript, we first quantify the DoTEOS functionalization, then elaborate on the measurements of *θ_app_*, and lastly discuss bijel synthesis with the functionalized nanoparticles.

ATEOS functionalization is realized on Ludox TM‐50 SNPs (20–30 nm). During the reaction at 70 °C in a solution of ethanol, water, and acetic acid, ATEOS undergo hydrolysis and condensation to covalently bind to silanol groups on the colloidal SNPs.^[^ ^36^ ^]^ To control %‐ATEOS functionalization, we vary the concentration of ATEOS reagents during the synthesis. Here, 100%‐ATEOS functionalization corresponds to the theoretical number of ATEOS molecules required to functionalize all initial silanol groups of the particles. We estimate the number of silanol groups with the specific surface area of the nanoparticles (140 m^2^ g^−1^) and assume an average amount of 4 silanol groups per nm^2^.^[^ ^37^ ^]^ After functionalization, the ATEOS‐SNPs are purified via 4 consecutive centrifugation/redispersion steps in water and 2‐propanol. We will provide the most results for dodecyltriethoxysilane functionalized SNPs (DoTEOS‐SNPs). Later, we will discuss additional results for SNPs functionalized with octyltriethoxysilane (OTEOS‐SNPs). In the next section, we characterize DoTEOS‐SNPs via thermogravimetric analysis (TGA).

**Figure 2:**
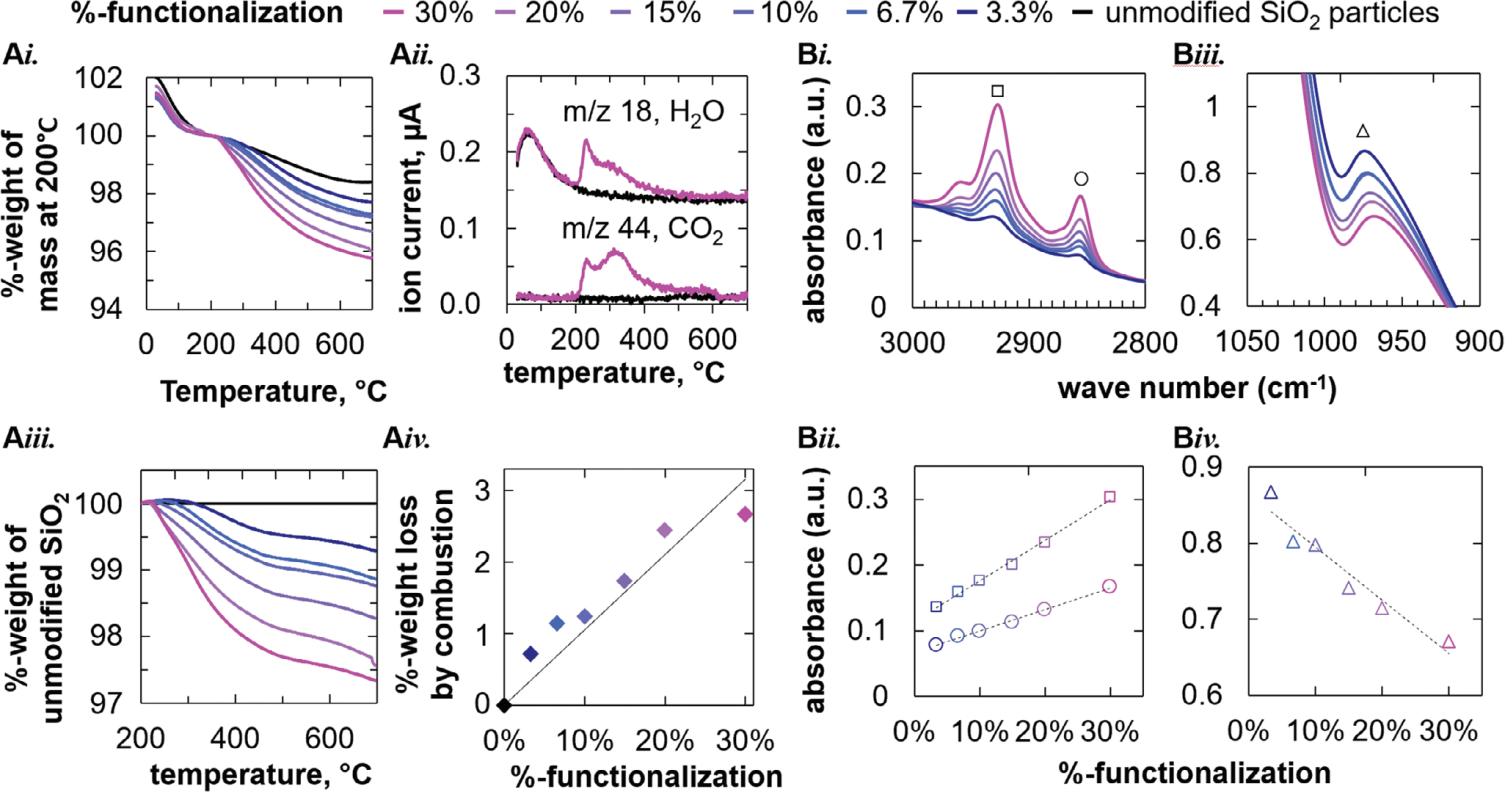
Analysis of the DoTEOS‐SNPs. A*i*) Thermogravimetric analysis (TGA) of DoTEOS‐SNPs. A*ii*) Mass spectrometry (MS) curves for SNPs with 30%‐DoTEOS functionalization and the bare/unfunctionalized SiO_2_ particles. A*iii*) %‐weight of DoTEOS‐SNPs normalized by the %‐weight of the unfunctionalized SNPs. A*iv*. %‐weight loss at 700 °C from A*iii*. B) Fourier‐transform infrared spectroscopy (FTIR) of DoTEOS‐SNPs.

TGA allows us to determine the amount of covalently bound DoTEOS on the SNPs. To this end, DoTEOS‐SNP powders are heated from room temperature to 700 °C. **Figure** **2**A *i*. shows the relative weights of the DoTEOS‐SNP samples (normalized with their respective masses at 200 °C) against the temperature.

Based on the TGA measurements, we can distinguish the mass reductions in two parts: *i*. the evaporation of physisorbed water and *ii*. the combustion of covalently bound hydrocarbons. From 30 to 200 °C, the %‐weights of all samples decrease equally with the temperature in Figure 2A *i*. During the TGA measurements, we also analyze the ionic currents of the formed gases via mass spectrometry (MS). MS detects a peak of water molecules with a characteristic mass‐to‐charge (m/z) ratio of 18 from 30 to 200 °C, as shown for 30%‐DoTEOS functionalization by the magenta top curve of Figure 2A *ii*. The same ionic current peak is observed for the unfunctionalized SNPs (black curve), suggesting the evaporation of physisorbed water from both unfunctionalized and DoTEOS functionalized particles. After plateauing ≈200 °C in Figure 2A *i*, the %‐weight decreases further for all samples. Interestingly, above 200 °C in Figure 2A *i*, larger %‐weight reductions are measured for DoTEOS‐SNPs with higher %‐DoTEOS functionalizations. For DoTEOS‐SNPs, the ionic currents show peaks for both H_2_O and CO_2_ (m/z ratio 44) in the temperature range from 200 to 700 °C (magenta curves in Figure 1A *ii*). In contrast, no generation of H_2_O, nor CO_2_ can be detected for the unfunctionalized SNPs particles from 200 to 700 °C (black curves of Figure 1A *ii*). We can conclude that the generation of H_2_O and CO_2_ from DoTEOS‐SNPs indicates the combustion of covalently attached alkyl moieties.

The weight reduction of the DoTEOS‐SNPs can be correlated to the %‐functionalizations controlled during the synthesis. In Figure 2A *iii*., the %‐weight of all samples is normalized by the %.weight of the bare/unfunctionalized SNPs. This normalization allows us to determine the %.weight loss resulting from the combustion of alkyl moieties on the SNPs. Figure 2A *iv*. shows that the %‐weight loss related to the combustion of alkyl moieties scales roughly linearly with the experimentally set %‐DoTEOS functionalization during the synthesis step. The TGA results allow us to estimate the % of added DoTEOS molecules that have covalently attached to the SNPs. To this end, we compare the %‐weight loss by combustion to the mass of dodecyl chains added during the functionalization step. We find that on average 88% of the added DoTEOS molecules have covalently bound to the SNPs.

Next, we identify the chemical changes on the particle surface via Fourier‐transform infrared spectroscopy (FTIR). To allow for quantitative comparison of particle spectra with variable %‐functionalization, the baseline is subtracted and the spectra are normalized to the Si─O─Si bending vibration peak at 800 cm^−1^ (assumed to remain unchanged by DoTEOS functionalization, Figure S1, Supporting Information). Figure 2B *i*. plots the normalized FTIR spectra of SNPs with variable %‐DoTEOS functionalization from 3000–2800 cm^−1^. Two characteristic peaks that grow in height with increasing %‐functionalization are observed. Both peaks are characteristic of alkanes and can be related to the dodecyl moiety of DoTEOS. Figure 2B *ii*. shows that both peaks increase linearly with the %‐functionalization, indicating increasing amounts of dodecyl chains on the particles. Figure 2B *iii*. plots the Si─OH peak at 975 cm^−1^.^[^ ^38^ ^]^ Interestingly, Figure 2B *iv*. shows that the Si─OH vibration decreases with the %‐functionalization. A decreasing Si─OH vibration suggests that hydrolyzed triethoxy moieties (Si─OH) of DoTEOS have undergone complete condensation to generate Si─O─Si bonds with SNP silanol groups and with other DoTEOS molecules. Complete Si─OH condensation follows from the fact that incomplete condensation would result in a growing Si─OH peak, because each hydrolyzed DoTEOS introduces two Si─OH groups if only one is bound to the nanoparticle. Thus, the combined TGA and FTIR analysis demonstrates the controllable attachment of dodecyl chains and the condensation of Si‐OH bonds.

**Figure 3:**
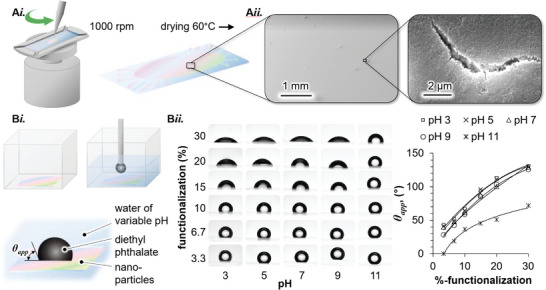
Contact angle measurements on spin‐coated DoTEOS‐SNP films. A*i*) Schematic of spin coating experiment. A*ii*) Scanning electron microscope images of spin‐coated DoTEOS‐SNP film. B*i*) Schematic of the experimental setup to measure the apparent contact angle *θ_app_*. B*ii*) Photographs of sessile droplets on spin‐coated DoTEOS‐SNP films of variable %‐functionalization and pH value of the surrounding aqueous phase. B*iii*) Measured values *θ*_app_ for variable %‐DoTEOS functionalization and pH value.

After quantifying the functionalization via TGA and FTIR, we next probe the effect of the dodecyl moieties on the SNP wettability. To prepare the wettability measurement, 2 wt‐% DoTEOS‐SNP dispersions in 2‐propanol of defined %‐DoTEOS functionalization are spin‐coated on glass slides, as schematically depicted in **Figure** **3**A *i*. Scanning electron microscopy analysis in Figure 3A *ii*. confirms that spin coating yields homogeneous coatings of DoTEOS‐SNPs on the glass slides after drying at 60 °C. The magnified inset exemplifies a microscopic crack in the DoTEOS‐SNP film, showing that the film consists of many layers of DoTEOS‐SNPs. We next measure apparent contact angles *θ*_app_ on the homogeneous and thick DoTEOS‐SNP coatings.

The setup to measure *θ*_app_ is schematically depicted in Figure 3Bi.^[^ ^39^, ^40^ ^]^ First, we submerge the spin‐coated and dried DoTEOS‐SNP films in water of specified pH values. Next, we deposit an oil droplet (DEP) on the DoTEOS‐SNP film. *θ*_app_ is measured through the water phase. We distinguish between *θ*_app_ of sessile droplets and *θ* of interfacial nanoparticles (Figure 1E) because: *i*) The measured *θ*_app_ is likely also influenced by the penetration of water or oil into the interstitial spaces between the DoTEOS‐SNPs, and thus only partially representative for the DoTEOS‐SNP surfaces. *ii*) For nanoparticles at interfaces, deviations from the Young equation are possible, for example, due to line tension effects.^[^ ^41^, ^42^ ^]^ *iii*) The drying step can change the surface chemistry of the DoTEOS‐SNPs.^[^ ^27^ ^]^ Nevertheless, *θ*_app_ provides insights into the trends of the nanoparticle wettability in dependence on the %‐DoTEOS functionalization, as discussed in the following.

Figure 3B *ii*. shows photographs of sessile droplets and a plot of *θ*_app_ in dependence of %‐DoTEOS functionalization and pH. In the range of pH 3 to pH 9, *θ_app_* decreases only slightly with increasing pH (e.g., for 30%‐DoTEOS functionalization from 130° ± 2.2 at pH 3 to 125° ± 1.5° at pH 9). In contrast, when the pH is further increased from pH 9 to pH 11 for 30%‐DoTEOS functionalization, *θ*_app_ decreases significantly to 72° ± 2.1°. The decrease of *θ*_app_ at pH 11 can be explained by the solubility of SiO_2_ above pH 10.^[^ ^43^, ^44^ ^]^

In contrast to the pH, the %‐DoTEOS functionalization has a strong effect on *θ_app_*. Increasing %‐DoTEOS functionalization from 3.3% to 30% increases *θ*_app_ from 44° ± 1.6° to 130° ± 2.2° at pH 3, and from 28° ± 1.7° to 125° ± 1.5° at pH 9. This observation confirms that the %‐DoTEOS functionalization allows for a gradual control over the wettability of the SNPs, important for bijel stabilization as discussed in the last section of this manuscript. Thus, from the *θ*_app_ measurements, it can be concluded that in the range of pH 3–9, the %‐DoTEOS functionalization has a strong effect on *θ*_app_, but the pH value influences *θ*_app_ only weakly.

**Figure 4:**
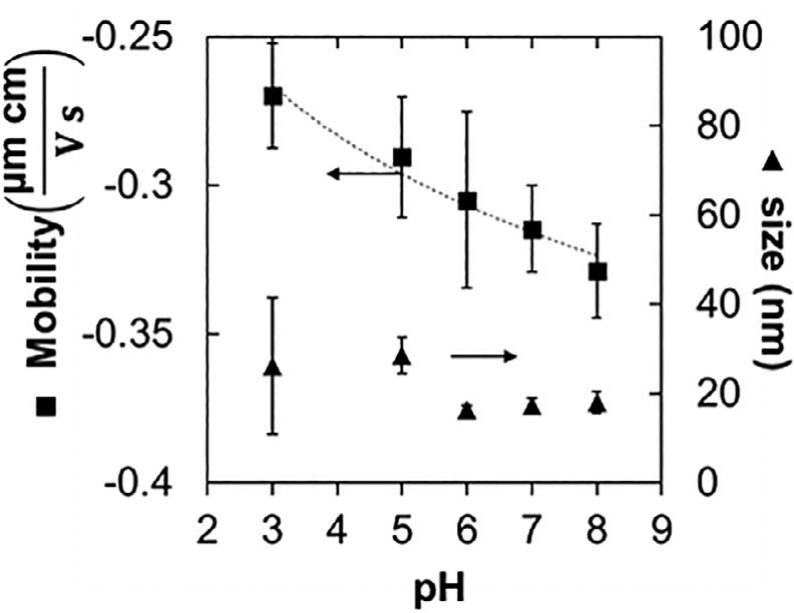
Electrophoretic mobility and dynamic light scattering of the particles with 10‐% functionalization in 2‐propanol.

Although the pH value does not appear to strongly change *θ*_app_, the pH has a significant effect on the electrophoretic mobility of the particles. After DoTEOS‐functionalization, the SNPs are purified via 3 consecutive centrifugation/redispersion steps in water. After the 3rd step, the pH value of all dispersions is measured to pH 3 ± 0.5, regardless of the %‐DoTEOS functionalization. We increase the pH value with 0.1 mol L^−1^ NaOH to pH 3, 5, 7, 8, and 9 and centrifuge the DoTEOS‐SNP dispersions. The DoTEOS‐SNP sediments are redispersed in 2‐propanol, and after that one more time centrifuged and redispersed in 2‐propanol. **Figure** **4** shows the results of electrophoretic mobility and dynamic light scattering measurements of DoTEOS‐SNPs with 10‐%‐functionalization in 2‐propanol against the last measured pH value in water. The average DoTEOS‐SNP size remains approximately constant ≈20 nm, although at pH 3 a wider size distribution from 17–40 nm is observed. However, the electrophoretic mobility decreases from −0.27 to −0.33 µm cm V^−1^ s^−1^ by increasing the pH value from 3 to 8. The decreasing electrophoretic mobility suggests that with increasing pH value, the amount of negatively charged silanol groups on the DoTEOS‐SNPs increases, also when dispersed in 2‐propanol.

**Figure 5:**
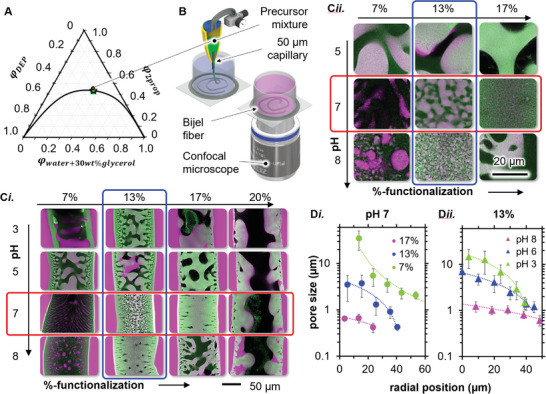
Bijel fiber synthesis with DoTEOS‐SNPs. A) Phase diagram (based on volume fractions *φ_i_*) with a binodal curve, a critical point (green star), and composition of bijel precursor mixture (red dot). B) Schematics of bijel fiber fabrication and confocal microscopy characterization. C*i*) CLSM images of equatorial planes of bijel fibers fabricated with different %‐DoTEOS functionalizations and pH values. *Cii*) Magnified CLSM images from selected samples of C*i* in the center of the fibers. D) Radial pore sizes in dependence of D*i*. the %‐DoTEOS functionalization and D*ii*. the pH value, lines are drawn to guide the eye.

After characterizing the DoTEOS‐SNPs, we test bijel formation with the dispersions of 30–40 wt.% DoTEOS‐SNPs in 2‐propanol. To this end, we employ a homogeneous bijel precursor mixture composed of water, glycerol, diethylphthalate (DEP), 2‐propanol, and DoTEOS‐SNPs. DEP is not miscible with water and glycerol. However, a miscible/homogeneous liquid mixture can be obtained by adding 2‐propanol.^[^ ^31^ ^]^ Glycerol is added to the mixture to increase the refractive index of the water phase, needed for confocal imaging of the bijel structures.^[^ ^45^ ^]^ To generate bijels with this mixture, we initiate phase separation via spinodal decomposition.^[^ ^26^, ^32^ ^]^ However, spinodal decomposition requires a mixture near the critical point of the phase diagram. We determine the critical point and the binodal curve experimentally via volumetric analysis and turbidimetry (Figure S2, Supporting Information). In the phase diagram of **Figure** **5**A, we plot the horizontal axis of the triangle as a mixture of water+30% glycerol. Interestingly, the binodal curves with and without glycerol overlap reasonably well, suggesting that glycerol has similar miscibility with DEP and 2‐propanol as water does (Figure S2, Supporting Information). We determine the critical point (green star) in volume fractions of 2‐propanol $\varphi_{2\mathrm{propi}}^{c}$ = 0.434, DEP $\varphi_{\mathrm{DEP}}^{c}$ = 0.208, water+30 wt.% glycerol $\varphi_{\mathrm{water}+30\mathrm{wt}.\%\;\mathrm{glycerol}}^{c}$ = 0.359. The approximate precursor compositions is depicted with the red dot in Figure 5A. Besides the liquids, each precursor mixture contains 16 wt.% of nanoparticles with a specified %‐functionalization.

With the precursor mixture, we generate bijel fibers via the experimental setup depicted in Figure 5B.^[^ ^45^ ^]^ The precursor mixture is pressurized at 1 bar and flown through a glass capillary of 50 µm inner diameter into a container of toluene. The diffusion of 2‐propanol to toluene results in a continuous fiber that sinks to the bottom of the vial, as schematically depicted in Figure 1B. The DEP in the bijel channels is exchanged through the interdiffusion of toluene, facilitated by the 1:1 miscibility of both liquids. The surfactant‐free bijels are stable in toluene for at least 8 h as shown in Figure S3 (Supporting Information). However, the bijel structures cannot be imaged in toluene via confocal laser scanning microscopy (CLSM) due to strong light scattering as shown in Figure S6 (Supporting Information). To improve CLSM imaging, we exchange toluene after bijel formation with hexane, which is also fully miscible with toluene. Figure S6 (Supporting Information) shows that in hexane complete imaging of the bijel interior is possible. Additionally, glycerol addition to the precursor mixture further enhances the imaging of the complete bijel structure as shown in Figure S4 (Supporting Information). Exchanging toluene with hexane can result in the disintegration of the bijels made with ATEOS‐SNPs. However, we find that stable bijels in hexane can be obtained upon dissolving 5 vol.% pentanol in n‐hexane and saturating this solution with cetyltrimethylammonium bromide (CTAB) and the fluorescent dye Nile Red. Thus, CTAB functionalization of the ATEOS‐SNPs enhances the stability in hexane, but CTAB is not necessary for the stability of the surfactant‐free, ATEOS‐SNP stabilized bijels in toluene.

We first focus on the effect of %‐DoTEOS functionalization of the SNPs on the bijel structures. Figure 5C *i*. shows CLSM images of the equatorial plane of fibers made with nanoparticles of variable %‐DoTEOS functionalization and pH values. The hexane phase is colored in magenta, the water phase in black, and the DoTEOS‐SNPs in green. By increasing the %‐functionalization at pH 7 (red box in Figure 5C *i*.), the structures transition from anisotropically aligned hexane and water macrovoids (7%‐functionalization), to a network structure reminiscent of spinodal decomposition (13%‐functionalization), to a dense structure with anisotropically aligned water cavities (17%‐functionalization), to coarsened structures with a large water cavity in the center (20%‐functionalization). The magnified insets in Figure 5C *ii* show that the dense structure at 17%‐functionalization consists of a fine microstructure of ATEOS‐SNP stabilized water/oil networks.

From the CLSM images, we measure the water pore sizes and plot the result in Figure 5D *i*. for the variation of %‐DoTEOS functionalization at pH 7. For 7%‐DoTEOS functionalization, the central water pores (radius 0–20 µm) are on the order of tens of micrometers and decrease to several micrometers toward the outer fiber radius. At 13%‐DoTEOS functionalization, the pore sizes in the fiber center are several micrometers in size and decrease to sub‐micrometer sizes toward the outer fiber radius. Remarkably, for 17%‐DoTEOS functionalization, the pore sizes are several hundreds of nanometers throughout the entire fiber radius. Interestingly, our measurements in Figure 3 detect *θ*_app_ = 90° for 17%‐DoTEOS functionalization.

The functionalization of the SNPs with octyltriethoxysilanes (OTEOS) enables similar control of the bijel structure as in Figure 5. However, in comparison to DoTEOS, OTEOS needs higher %‐functionalizations of 25–35% to generate network structures of micrometer pore sizes (Figure S5, Supporting Information). Thus, both the OTEOS‐SNPs and DoTEOS‐SNPs enable the formation of surfactant‐free bijels.

Importantly, also the pH values of the ATEOS‐SNP dispersions in water before transfer to 2‐propanol control the structures. Figure 5C *i* illustrates that with a 13%‐DoTEOS functionalization (indicated by the blue box in Figure 5C *i*.), network structures exhibiting a pronounced pore size gradient across the fiber radius are observed at both pH 3 and pH 5. The pore sizes become significantly more uniform as the pH is increased to pH 7 at 13%‐DoTEOS functionalization. The trend of decreasing pore gradient persists at pH 8 for 13%‐DoTEOS functionalization, where a dense structure with anisotropically aligned water cavities is observed. The magnified insets in Figure 5C *ii* reveal that this dense structure also comprises a fine microstructure of DoTEOS‐SNP stabilized water/oil networks.

The radial pore size analysis is in Figure 5D *ii*. shows quantitatively how increasing the pH value decreases the pore sizes from 10 µm at pH 3 into the sub‐micrometer range at pH 8. The strong effect of the pH value on the bijel structures is remarkable, because the pH value has no significant influence on *θ_app_*, as observed in Figure 3B *ii*. Thus, in contrast to the pore size trend in dependence of %‐DoTEOS functionalization, the different structures at variable pH seem to have other reasons than changes of *θ*_app_. The pH affects primarily the particle ionization as shown by the electrophoretic mobility measurements in Figure 4. Further research is required to elucidate the mechanisms and dynamics behind STRIPS‐bijel formation in dependence on the ATEOS‐SNP ionization.

Before concluding, we discuss similarities of the present bijel system compared to prior work. We found that ATEOS‐SNPs provide long‐term stability in toluene, but limited stability in hexane. This observation is similar to what was previously found for bijels made with hexamethyldisilazane (HMDS) functionalized particles, using ethanediol and nitromethane (ED/NM).^[^ ^28^ ^]^ The ED/NM bijels made with HMDS functionalized particles also change their structure when NM is exchanged with other oils.^[^ ^29^ ^]^ In contrast, the bijel system composed of water/lutidine networks allows for exchanging the oil phase without structural disintegration.^[^ ^17^, ^29^ ^]^ The stability of the water/lutidine bijel has been related to attractive van der Waals interactions between the silica particles.^[^ ^46^ ^]^ Prior work has also shown that CTAB functionalized particles enable stable bijels after changing the oil phase, suggesting that also surfactant‐containing bijels can be stabilized via attractive forces between the particles.^[^ ^26^ ^]^ Thus, it appears that the stability of bijels made with ATEOS‐SNPs in toluene is primarily based on interfacial tension‐driven jamming of the ATEOS‐SNPs, rather than attractive ATEOS‐SNP interactions. However, further research is needed to comprehend the interfacial stabilization mechanisms, for example via rheological characterization of ATEOS‐SNP decorated interfaces.

## 3 Conclusion

In conclusion, this work demonstrates for the first time how bijels with sub‐micrometer domains can be synthesized with alkyltriethoxysilane (ATEOS) functionalized silica nanoparticles (ATEOS‐SNPs), while prior research required surfactants to generate nanostructured bijels.^[^ ^24^, ^26^, ^33^ ^]^ The acetic acid‐catalyzed ATEOS functionalization of the SNPs enables precise control over the number of attached alkyl moieties, enabling gradual control over the wettability. Moreover, adjustment of the pH value enables control over the surface ionization of the particles. Our results show that the ATEOS‐SNP wettability is primarily controlled via the %‐ATEOS functionalization, and to a lesser extent by the nanoparticle ionization. We find an optimum for the number of covalently attached ATEOS groups and SNP ionization, enabling the formation of bijels with sub‐micrometer domains. With this knowledge, bijel synthesis becomes more accessible to researchers from different disciplines, promoting the exciting application potentials of bijels as nanomaterials with tunable and functional attributes. Future research on bijel synthesis with covalently functionalized particles needs to investigate bijel stability in different liquids,^[^ ^29^ ^]^ the mechanisms and dynamics behind bijel formation, the use of other functional groups besides alkyl chains,^[^ ^30^ ^]^ and the potentials to generate bijels via direct mixing.^[^ ^23^, ^24^ ^]^ ATEOS‐SNPs can also enable new bijel postprocessing routes into functional nanomaterials for applications in catalysis,^[^ ^22^ ^]^ energy storage,^[^ ^19^ ^]^ membrane separations,^[^ ^20^ ^]^ or healthcare materials.^[^ ^47^ ^]^

## Conflict of Interest

The authors declare no conflict of interest.

## Supporting information

Supporting Information

## Data Availability

The data that support the findings of this study are available from the corresponding author upon reasonable request.
