# Supplementary material for: Stabilizing Bicontinuous Emulsions with Sub‐Micrometer Domains Solely by Nanoparticles
Source: Adv Sci (Weinh). 2024 Aug 20;11(39):2406223. doi: 10.1002/advs.202406223 (PMC11497009; doi:10.1002/advs.202406223)
Supplement: Supplementary file 1 — Supporting Information [file ADVS-11-2406223-s001.pdf]

ADVANCED SCIENCE
Open Access

Supporting Information

for *Adv. Sci.*, DOI 10.1002/advs.202406223

Stabilizing Bicontinuous Emulsions with Sub-Micrometer Domains Solely by Nanoparticles

*Alessio J. Sprockel, Tessa N. Vrijhoeven, Henrik Siegel, Ffion E. Steenvoorden and Martin F. Haase**

Supporting information

Stabilizing Bicontinuous Emulsions with Sub-Micrometer Domains Solely by Nanoparticles

Alessio J. Sprockel, Tessa Vrijhoeven, Ffion Steenvoorden, Henrik Siegel, Martin F. Haase

Contents

1 Materials and methods 2
 1.1 Materials 2
 1.2 Particle modification 2
 1.3 Precursor preparation and bicontinuous structure analysis 3
 1.4 FT-IR analysis 3
 1.5 TGA analysis 3
 1.6 DLS analysis 3
 1.7 Spin coating procedure and contact angle analysis 3
2 FTIR spectra Dodecyltriethoxysilane 4
3 Measurement of the ternary phase diagram 5
4 Time dependent stability of bijel 5
5 Confocal comparison effect of glycerol addition 6
6 Confocal data of octyltriethoxysilane 7
7 Confocal data of octyltriethoxysilane 7

1 MATERIALS AND METHODS

1.1 Materials

All water used is ultrapure water (Type I water) purified using a Reptile Genie U12 system. All chemicals were used as received.

Table 1 Chemicals utilized

Material	Purity	Supplier
Hydrochloric acid	37%	Acros
Potassium Bromide (IR)	99+%, IR grade	Acros
Dodecyltriethoxysilane	technical	Aldrich
DEP	99%	Alfa Aesar
Hexane	HPLC grade	Biosolve
Acetic acid	≥99.7%	Fisher
2-propanol	for HPLC, ≥99.9%	Honeywell
Ethanol	Absolute for analysis	Merck
Sodium hydroxide	Pellets for analysis	Merck
Potassium hydroxide	Pellets for analysis	Merck
CTAB	≥99%	Sigma
Ludox TM-50	50 wt.%	Sigma-Aldrich
Nile Red	for microscopy	Sigma-Aldrich
1-pentanol	99%	Sigma-Aldrich
Toluene	99+%	Thermo scientific

1.2 Particle modification

A PEG bath was placed on a hotplate and pre-heated to 70 °C. Ethanol (36.6 g) and Acetic acid (18.5 g) were added to a 100 mL glass bottle and shaken. Ludox TM-50 (22.4 g, 50 wt.% dispersion in water), and various amounts of dodecyltriethoxysilane were added under magnetic stirring, in that order. The bottle was closed, placed into the PEG bath, and left at 70 °C overnight with magnetic stirring. The amount of dodecyltriethoxysilane added is calculated by assuming a specific surface area of 140 m^2/g, and 4 silanol groups per nm^2. With these assumptions a modification of 10% supplies one silane molecule for each 10 silanol groups.

Table 1.1. *Amount of Dodecyltriethoxysilane added to functionalize 22.4g of Ludox TM-50.*

**Functionalization (f.%)**	3.3	6.6	10	15	20	30
**Dodecyltriethoxysilane (mL)**	0.132	0.264	0.396	0.594	0.792	1.188

Heating and stirring were stopped and the bottles were removed from the PEG bath. The mixture was transferred to a 250 mL centrifugation bottle and was washed by centrifugation (250 mL water, 3750 rpm, 10-90 minutes) three times. The range in centrifugation time stems from the varying colloidal stability of the particles. With higher functionalization the particles become more hydrophobic and unstable in water, thus less centrifugation is required. Where 10 minutes is sufficient for the highest functionalization, 90 minutes are required for the lowest. For redispersion, a combination of sonication, shaking, and stirring was employed. The dispersions were redispersed into 120 mL water and split into 40 mL portions for pH adjustment. Starting from approximately pH 3.6, sodium hydroxide (0.01 and 0.1 M) was added to adjust the pH upward, hydrochloric acid (0.01 and 0.1 M), was added to adjust the pH downward. The dispersions were separated by ultracentrifugation (13000 rpm, 10-90 minutes), and redispersed into 30mL 2-propanol. The dispersions were separated by ultracentrifugation one last time (75 000 g, 90 minutes) and redispersed into various amounts of 2-propanol. The particle weight fraction of the dispersions was determined by drying a weighed amount of dispersion, and adjusted to 35 wt.% by addition of 2-propanol.

1.3 Precursor preparation and bicontinuous structure analysis

DEP (0.480 mL), modified particles in isopropanol (0.960 mL, 35 wt.%), and 2-water (0.560 mL) were mixed in a 2 mL glass vial. Droplets of 2-propanol (approximately 0.03 mL) were added until the mixture turned clear after vortex agitation. A 50 µm capillary was glued into a 1 mL pipette tip and 0.1 mL of precursor mixture was transferred into the device. The pipette tip was attached to a pressure hose and using a pressure of approximately 1 bar, the precursor mixture was extruded into an imaging container filled with toluene. The toluene is carefully replaced with imaging medium (Hexane with 4 v.% pentanol, saturated with Nile red and CTAB), and the imaging container is placed onto the stage of a Leica Stellaris 5 confocal microscope. A z-stack of images (4-megapixel) is acquired at steps of 5 µm. The two imaging channels are configured as follows;

Table 1.2. *Confocal imaging parameters.*

Channel name	Color	Excitation	Collection
Oil	Magenta	488 nm	590 – 670 nm
Particle	Green	561 nm	630 – 800 nm

1.4 FT-IR analysis

250 mg potassium bromide was dried overnight at 80 °C. 200 µL modified particle dispersion (1 wt.% in 2-propanol) was added and the mixture was dried overnight at 80 °C. The mixture was transferred into a mortar, ground to a fine powder, and pressed into a transparent pellet (13 mm) using a hydraulic press (10 tons, 2 minutes). For each pellet three FTIR spectra (400-4000 cm^{-1}, 4 cm^{-1} step) were collected and averaged with a KBr pellet FT-IR spectrometer (PerkinElmer). The baseline was subtracted, and the spectrum was normalized using the Si-O-Si peak at 800 cm^{-1}.

1.5 TGA analysis

100 mg modified particles were pre-dried overnight at 80 °C. Measurements performed by Dennie Wezendonk on a TA Instruments 5500. Approximately 6 mg of sample is heated to 700 °C at a rate of 10 °C min^{-1} in a 20% O_2 in argon atmosphere. The sample mass is recorded, while the evolved gasses are analyzed using mass spectrometry.

1.6 DLS analysis

1 mL of modified particle dispersion (1 wt.% in 2-propanol) was prepared and pipetted into a Malvern DTS1070 cuvette with gold-plated electrodes. Using a Malvern Ultra, the zeta potential was determined, through electrophoretic light scattering, five times and averaged. The volume average size was determined, through dynamic light scattering, 3 times, and averaged. The measurements were performed at 25 °C.

1.7 Spin coating procedure and contact angle analysis

Microscopy slides (26x75x1mm) were cleaned using non-dusting wipes with ethanol. The slides were placed into a container filled with ethanol and sonicated, rinsed with new ethanol, and left in a base bath solution (20 g/L sodium hydroxide, 20 g/L potassium hydroxide) overnight. The slides were thoroughly rinsed with water, thoroughly rinsed with ethanol, and finally dried at 60 °C. For each sample, the slide is placed into a 3D-printed, lab-made, holder attached to an electrical spindle motor. 100 µL of modified particle dispersion (2 wt.% in 2-propanol) was prepared and dispensed onto a spinning (1000 rpm) slide over approximately 3 seconds. After 30 seconds the motor was powered down. The slide is placed into a holder and dried at 60 °C for 2 hours.

Water is prepared at various pH points using HCl and NaOH and placed into a glass container (90x40x40 mm). The spin-coated slide is placed into the container and the container is placed on the stage of a Dataphysics OCA25 system. A droplet of 8 µL DEP is placed onto the slide using the dosing system and the droplet is imaged using an IDS-Imaging CMOS 2-megapixel camera. 9 droplets are captured per sample and the contact angles are determined in ImageJ using the freely available contact angle plugin.[36]

2 FTIR spectra Dodecyltriethoxysilane

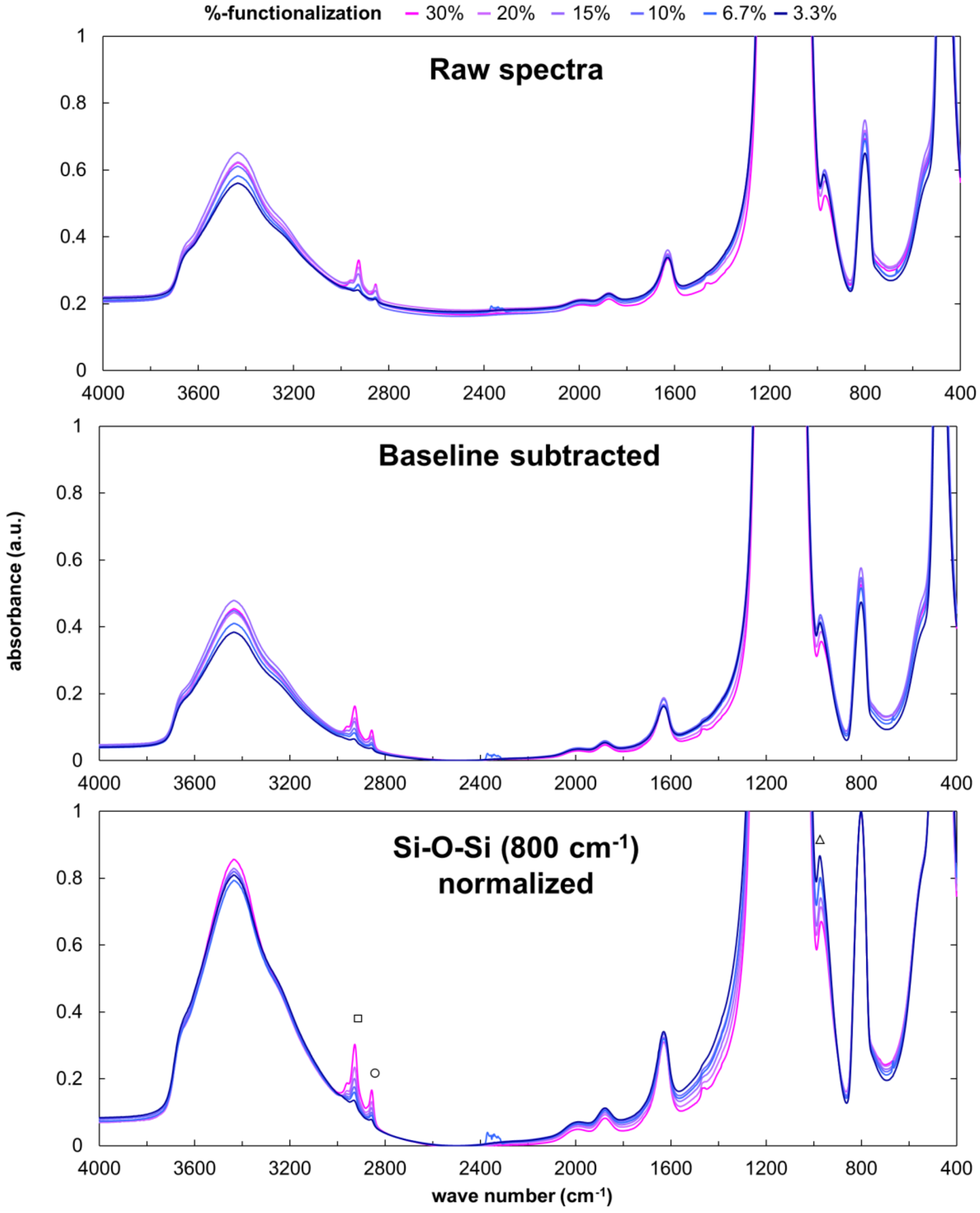

Figure S1. Complete FTIR spectra, and normalizing procedure of particles functionalized with DDTEOS.

For the normalization of the spectra with the Si-O-Si peak (800 cm^{-1}), we assume that the number of Si-O-Si bonds in the bulk of the silica nanoparticle is much larger than the Si-O-Si bonds generated by hydrolysis and condensation of DDTEOS molecules on the particle surface. Approximately 2% of all Oxygen of the nanoparticle is located on the surface, assuming 4 SiOH/nm^2 and a bulk density of 2.6 g/cm^3. DDTEOS functionalization adds a significantly smaller amount than these 2% to the surface, justifying the normalization procedure employed here.

3 MEASUREMENT OF THE TERNARY PHASE DIAGRAM

To determine the ternary phase diagram of DEP, IPA and water (with 30 wt-% glycerol), twelve binary mixtures were prepared by weight, marked by the purple squares on both sides of the ternary phase diagram. For each composition the third phase is then added dropwise and vortexed. Once the mixture becomes cloudy, the sample is weighed again to determine the demixing point (red circle). Now IPA is added until the sample mixes, and the sample is weighed to determine the mixing point (green circle). Thus, for each composition a path is travelled as is described by the black arrows for starting composition 62 vol-% DEP, 38 vol-% IPA. The binodal is taken to lie between the mixing and demixing points.
The critical point is determined by preparing mixtures just below the binodal. Here, for any mixture to the left or right of the critical point one phase is dramatically larger in size than the other. Very close to the critical point, the phases are equal in size.

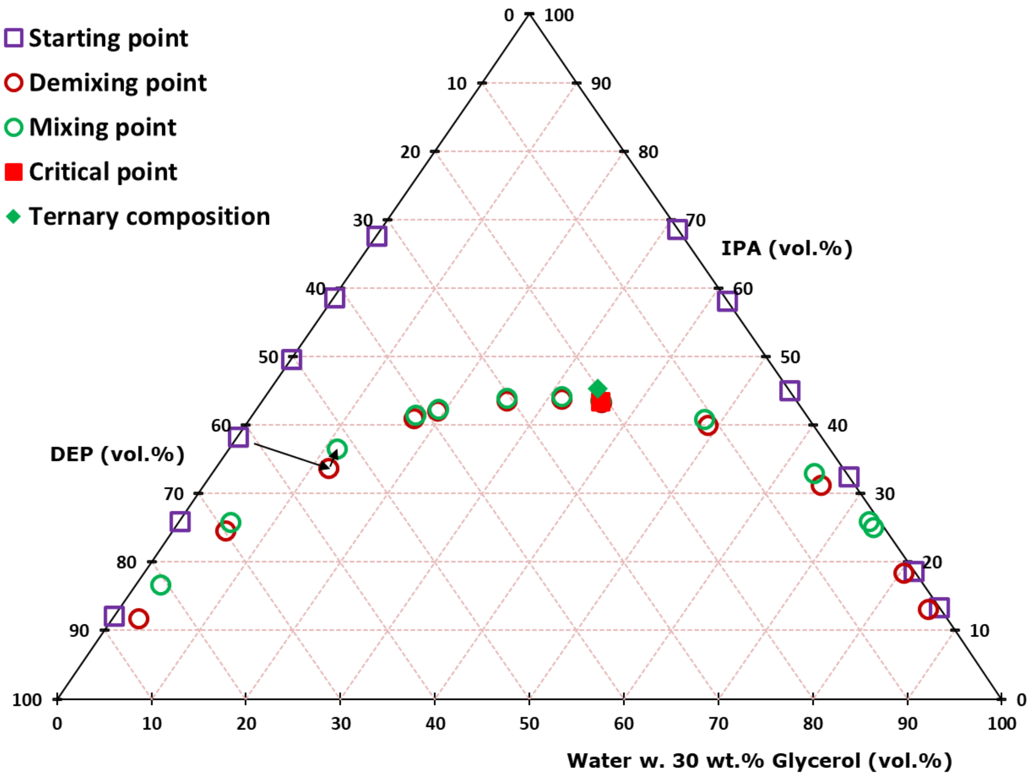

Figure S2. Measurement of the phase diagram

4 TIME DEPENDENT STABILITY OF BIJEL

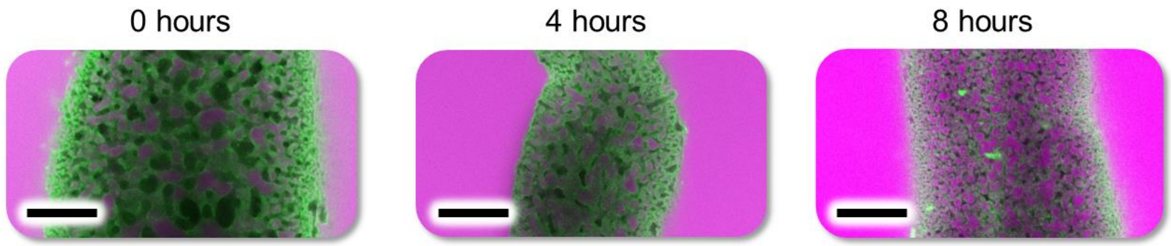

Figure S3. Bijel fiber stability. The fibers have been stored in toluene (water-saturated) for the amount of hours indicated above each image. After the indicated time, the toluene has been exchanged by hexane (5 vol-% pentanol saturated with CTAB). The fibers in the micrographs have been fabricated with octyltriethoxysilane functionalized Ludox TM particles, %-functionalization: 0.3, last measured pH value in water before transfer to 2-propanol: pH 7

5 CONFOCAL COMPARISON EFFECT OF GLYCEROL ADDITION

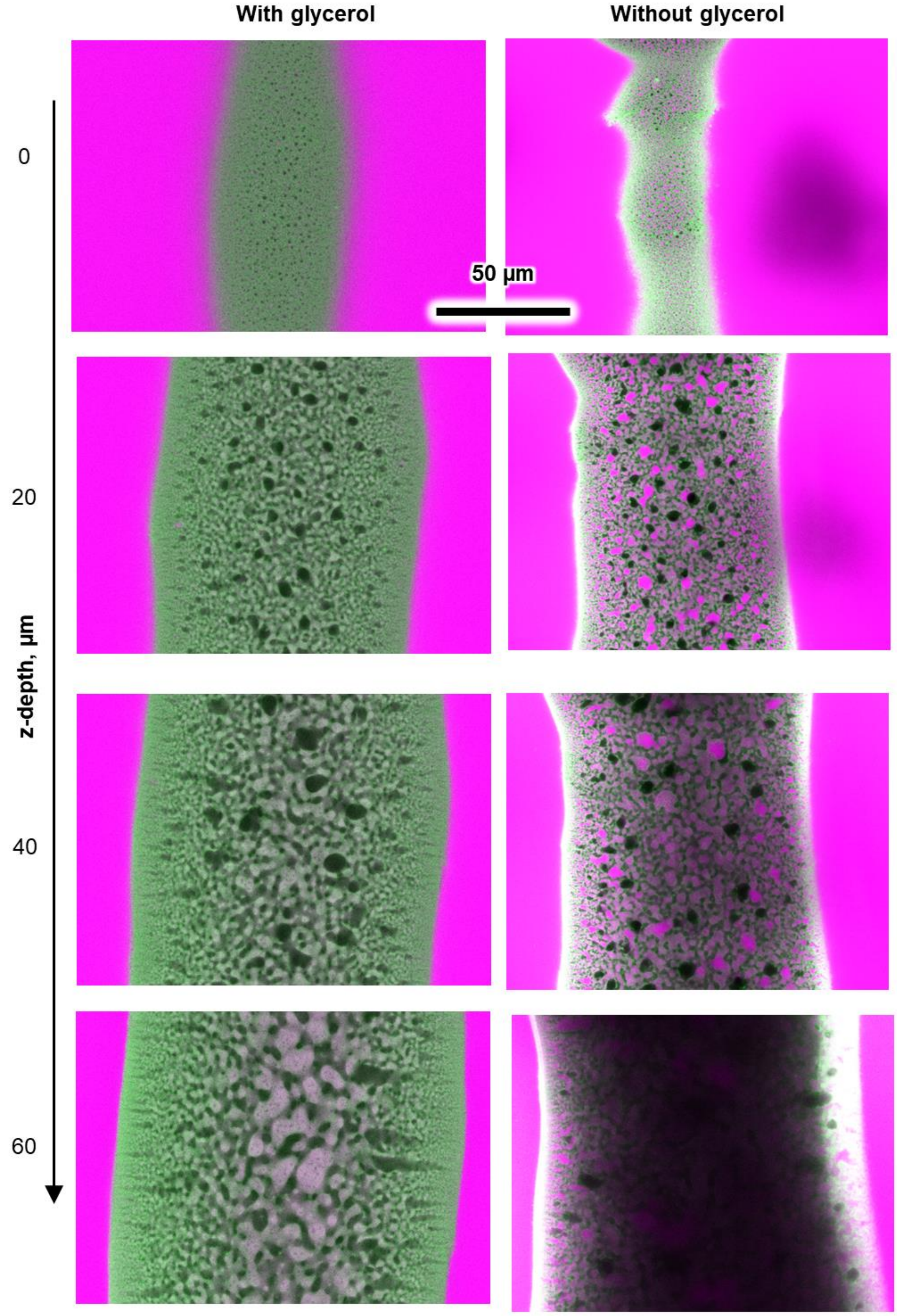

Figure S4. Comparison of bijel with/without glycerol

6 Confocal data of octyltriethoxysilane

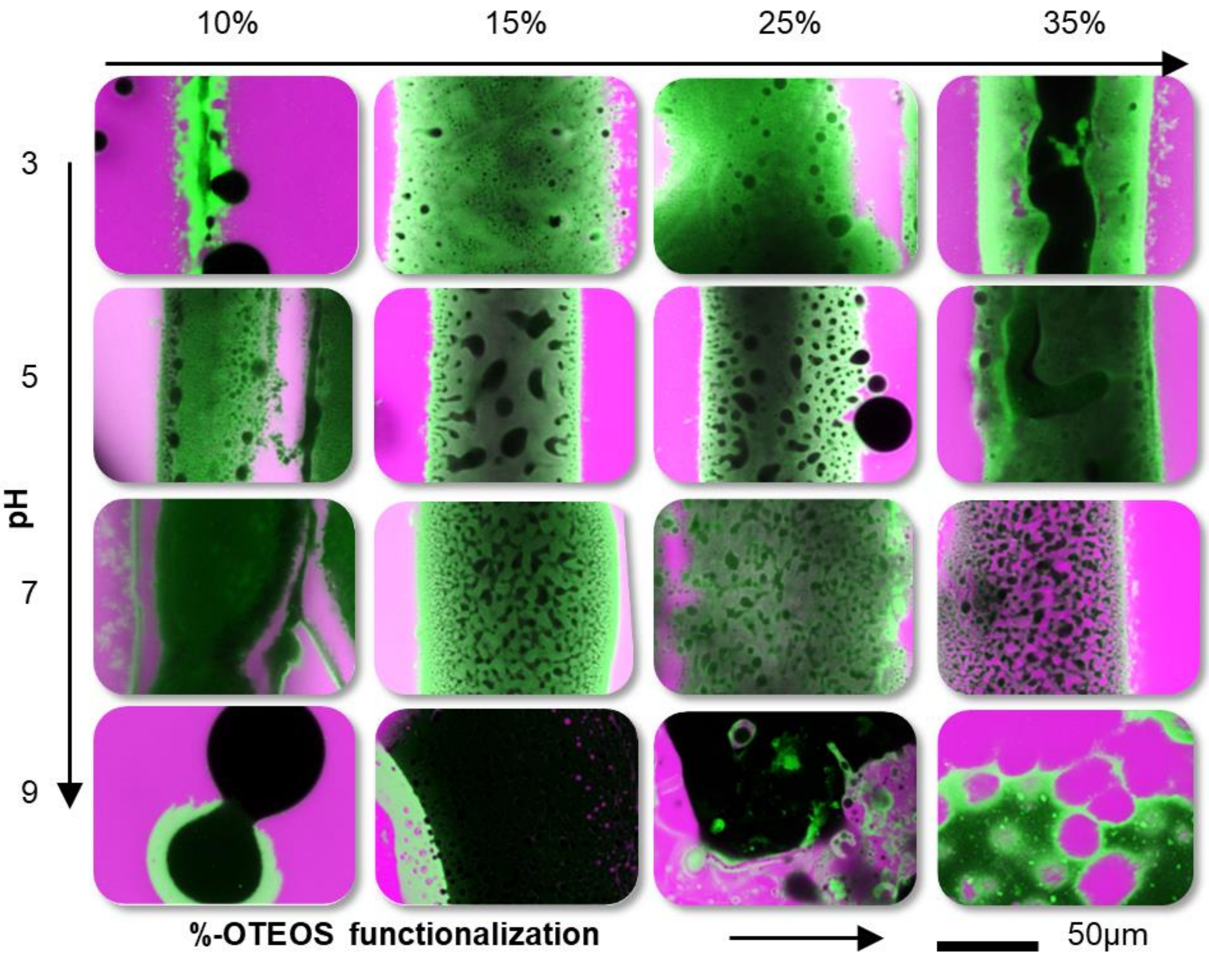

Figure S5.Confocal laser scanning microscopy images of fibers prepared with octyltriethoxy-silane (OTEOS) functionalized silica nanoparticles of variable %-OTEOS functionalization and pH values of the aqueous dispersion before transfer to 2-propanol.

7 Confocal data of octyltriethoxysilane

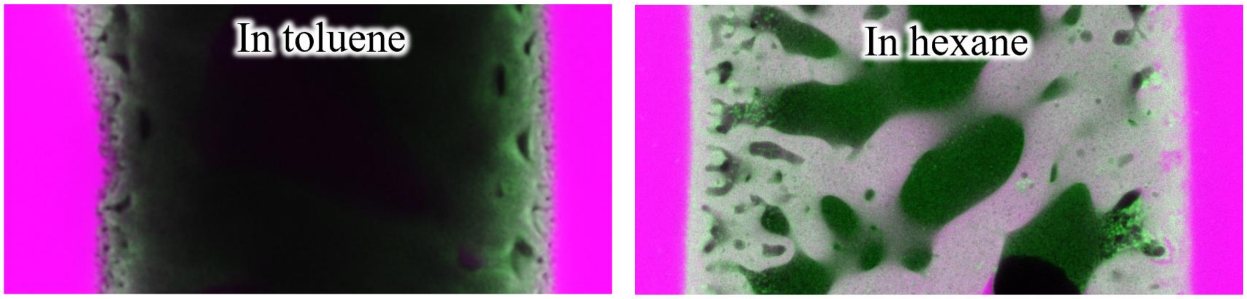

Figure S6. Confocal laser scanning microscopy images of bijel fibers using Nile Red as fluorescence dye in toluene and in hexane (5 vol-% pentanol and saturated with CTAB).
